# Essay on Crown Cavities in Molar Teeth

**Published:** 1860-01

**Authors:** Geo. L. Paine


### DENTAL REGISTER OF THE WEST.

Vol. XIII.] JANUARY, 1859. [No. 7.

### Original Essays and Communications.

### ESSAY ON CROWN CAVITIES IN MOLAR TEETH.

Read at the 1st Regular meeting of the Mad River Valley Dental Association, held in Springfield, November 17th, 1859.

BY GEO. L. PAINE.

By crown cavities, we mean those that are found in the grinding surfaces of the molar and bicuspid teeth. The decay generally commences in the depressions, when the enamel is congenitively defective.

We will take for illustration a small cavity in a first superior molar, which may be opened with a drill, which will, in many cases, remove all the decay. If, however, this end can not be attained by the use of this instrument, recourse must be had to others, such as excavators, with which every particle of decomposed dentine may and should be removed, prior to the introduction of the filling. The orifice of the cavity should be rendered smooth and slightly countersunk, rather than undercut, in order that a filling solid, and durable, may be inserted, and the preservation of the tooth thus secured at the point filled. The cavity being shaped to our liking, we may next describe our process of filling it with gold.

For the sake of being definite, we will describe our process of using soft foil, No. 4 or 6. We fold our sheet of gold into 4 or 6 thicknesses, cutting it into strips a little wider than the cavity is deep, next rolling on a 3 or 5 sided broach, these strips into cylinders, smaller in diameter than the cavity, making them successively smaller and harder.

Having washed out and dried the cavity, and adopted such precautions as will secure from moisture or saliva, the tooth to be filled, we will select one of the largest cylinders, and with our plugging pliers place it against the posterior wall of the cavity, and finally push it home, being careful that it maintain its position without change. We continue to introduce our cylinders against the wall of the cavity, condensing them as we proceed, till it is full; inserting the last, hardest and smallest in the center, thus rendering solid every part of the filling, from its circumference to its center. Lest our filling be not as dense as it should be, we will take a wedge or cone-shaped instrument, and force it into the body of the filling at the most yielding point and there introduce another hard cylinder, or adhesive gold—which we prefer to finish with. We next pass over the whole surface of the filling, a serrated condenser, in order to reduce irregularities, and consolidate as much as possible. The face of the filling may now be dressed down with a plug file, until level with the surface of the tooth, care being taken that there be no portions of the plug overlapping the tooth, as a nucleus for future decay. All the file marks may next be erased by rubbing the surface of the filling with a piece of Scotch stone, or a properly shaped piece of wood, carrying pulverized pumice stone and water, and after being washed to remove all grittings, it should be finished with a burnisher, occasionally dipped in a solution of castile soap. If our manipulations have throughout been conducted carefully and honestly, the result of our labor will be a substantial filling, which will resist the friction of mastication as effectually as solid and healthy enamel itself.

SECOND MODIFICATION.—In superior molars we often find a large cavity in the body of the tooth, while the orifice is small. We will, in this case, cut down with a gouge, the edges of enamel overhanging the cavity, in order to exhibit, and render accessible for removal, the decay, and secure for the border of the cavity a solid wall. We prefer to give our cavity a circular shape, avoiding, as much as possible, irregular shapes and acute angles under cuts, and ragged or brittle margins. The mode of filling is the same as described under the first modification.

THIRD MODIFICATION.—When it is impracticable to form a cavity cylindrical in shape,—but which has, as is sometimes the case, unavoidable undercuts, we must shape our blocks or cylinders so as to fill in these undercut portions, first making them as solid as possible, before we introduce our longer blocks to fill up the body of the cavity. Unless these undercut portions are well filled, the tooth will, ere long, break in, and require re-filling.

FOURTH MODIFICATION.—Crown cavities in second molars. (Superior.) In these teeth, we often find two cavities, which when the caries is all removed therefrom, there will be left between them only a thin partition of healthy bone. In cases of this kind, we prefer to cut this away entirely, and make of the two but one cavity. In these cavities, especially the posterior one, we find on the part of the caries, a disposition to run out into acute angles, following the sections found in the grinding surfaces of these teeth.

This decay must be followed up, no matter how angular our cavity may be, because the integrity of the wall must not be sacrificed to secure a cylindrical shape for it. In a word, that course must be pursued which will be most likely to secure the preservation of the tooth. There are other modifications peculiar to this class of teeth—such as anterior and posterior approximal, and buccal and palital, cavities running into crown cavities.

But as it does not come within our province to treat of these, we will turn our attention to crown cavities in inferior molars. We find, on examining these teeth, that the decay has a tendency to follow up the sutures, radiating from the central cavity, often involving the whole crown, insomuch that in some cases a large portion of the dentine must be cut away to arrest the progress of decay, and save the tooth,—hence large fillings are more frequently required in those teeth than in the superior molars.

Another modification of decay in these teeth is when there are divergent lines of decay. These lines are sometimes but slightly decayed, yet at their extremities, there often are pits, which are to be enlarged with a drill, the decay in the in the sutures or lines, removed, and the central cavity prepared in the usual manner. The pits are first to be filled, following up the sutures and ending in the central cavity. We regard it as wholly unsafe to leave a decayed suture unfilled, however slightly it may be effected—because neglect here, is fatal, not only to the crown cavity filled—but to the tooth itself.

